# Role of Polymeric Stabilizing Agents as a Molecular Spacer in Gold Nanoparticle-Mediated FRET-Based Biosensing

**DOI:** 10.3390/bios15090593

**Published:** 2025-09-09

**Authors:** Atul Kumar Tiwari, Roger J. Narayan

**Affiliations:** 1 Department of Chemistry, Indian Institute of Technology (BHU), Varanasi 221005, India; 2 Joint Department of Biomedical Engineering, University of North Carolina, Chapel Hill, NC 27695, USA

**Keywords:** FRET, molecular spacer, synthetic polymers, gold nanoparticles, biosensing

## Abstract

In FRET-based sensing, the interaction between the analytes and metal nanoparticles is significantly regulated by the physicochemical characteristics of the nanoparticles, such as their shape, size, zeta potential, surface-linked ligands, doping, pH of the medium, particle surface roughness, and lattice structure (atomic arrangements). During the synthesis process, to avert the aggregation of gold nanoparticles (AuNPs), synthetic polymers (including polyethylene glycol, polyethyleneimine, and poly-N-vinylpyrrolidone) and natural polymers (such as chitosan, starch, gellan, welan, and κ-carrageenan) are frequently employed for stabilization. This stabilization is accomplished through mechanisms such as steric repulsion and electrostatic stabilization, which form a protective layer around AuNPs. These stabilizing polymers act as molecular spacers in nanoparticle-based FRET sensing, enabling the precise regulation of the molecular distance between the acceptor and donor fluorophore molecules. This regulation enhances the efficiency and sensitivity of FRET assays. By modifying the length and flexibility of the spacer polymer, researchers can adjust the spacing between fluorophores, ensuring effective energy transfer and the accurate detection of target molecules. However, there is a limited understanding of the role and broad application of these molecular spacers in nanoparticle-mediated FRET-based sensing of various analytes. Consequently, this review explores different fundamental aspects of FRET, polymeric stabilization of gold nanoparticles, and various polymeric spacers in FRET-based sensing, along with the recent advancements and challenges associated with this approach.

## 1. Introduction

**Figure 1:**
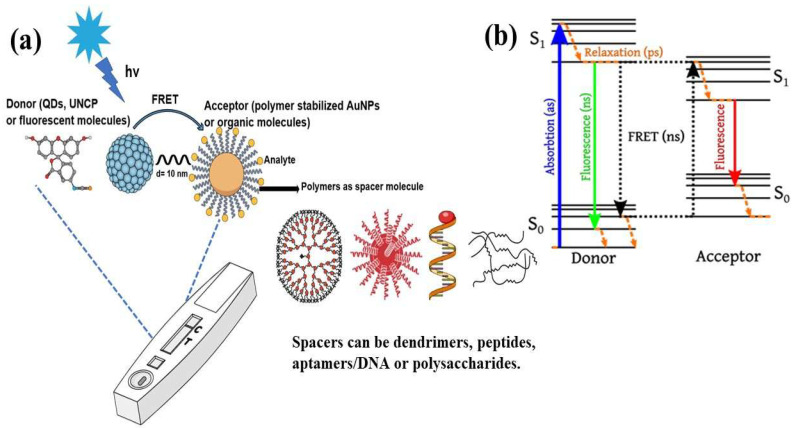
(**a**) Typical FRET-based biosensor diagram and (**b**) Jablonski diagram of FRET. Adopted under the Creative Commons Attribution-Share Alike 3.0 Unported license from ref. [6].

In analytical research, the detection of drug molecules, biological molecules, and various chemicals at extremely low concentrations is an important consideration. Fluorescence resonance energy transfer (FRET)-based sensors and biosensors, particularly those that utilize fluorescent probes, have proven effective in addressing these challenges. FRET has become a leading spectroscopic technique for detecting a variety of analytes at picomolar and nanomolar levels of sensitivity [1]. This technique allows for the precise and sensitive observation of biomolecules without the need for direct labeling or modification. FRET biosensors are noted for their high specificity, enabling them to identify target biomolecules without interference from other substances owing to their strong binding affinity and the engineered proximity between the donor and acceptor [1]. Their sensitivity, often reaching the picomolar range, is vital for detecting trace amounts of biomolecules, which is essential for the diagnosis of early disease and the monitoring of environmental pollutants. FRET biosensors can detect a wide variety of biomolecular and environmental changes, making them useful for applications, including monitoring protein–protein interaction, detecting pH levels, and monitoring enzymatic activity. This functionality of FRET biosensors makes them useful for medical diagnosis, biochemical monitoring, cell biology research, and drug discovery research. The potential applications of FRET-based biosensors include physiological monitoring, medical diagnosis, and environmental studies [2]. FRET is a physicochemical phenomenon based on the distance between two fluorophore molecules [1]. It involves quantum mechanical and non-radiative energy transfer without photon emission via dipole–dipole coupling from an excited donor to an electron acceptor in its ground state [1]. Essentially, energy is transferred through space rather than through the typical emission and subsequent reabsorption of light emitted by the donor. This transfer is only possible when the donor and acceptor molecules are physically separated by 1–10 nm, with overlapping electronic energy levels and appropriate fluorophore molecular orientation [2,3,4,5]. The Jablonski diagram in Figure 1 illustrates a donor–acceptor pair undergoing FRET, indicating the energy-transfer processes [6]. It depicts different energy levels: the ground state (S_0_), first excited singlet state (S_1_), first excited triplet state (T_1_), and non-fluorescent ground state (S_0_) after emission. Upon light absorption, the donor molecule transitions to S_1_. From this state, it can return to S_0_ through spontaneous emission, resulting in fluorescence or FRET occurring. During FRET, energy transfers from the excited donor state (S_1_) to the ground-state acceptor state (S_0_), which subsequently moves to S_1_. The donor then returns to S_0_ without emitting a photon, whereas the acceptor returns to S_0_ through photon emission [6].

**Figure 2:**
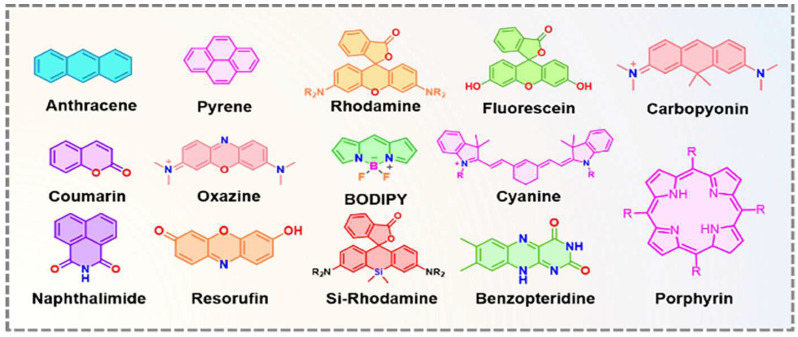
Some of the small fluorophore molecules used as donors, acceptors, or both in conventional FRET-based biosensor development. Adopted with permission from ref. [12].

**Figure 3:**
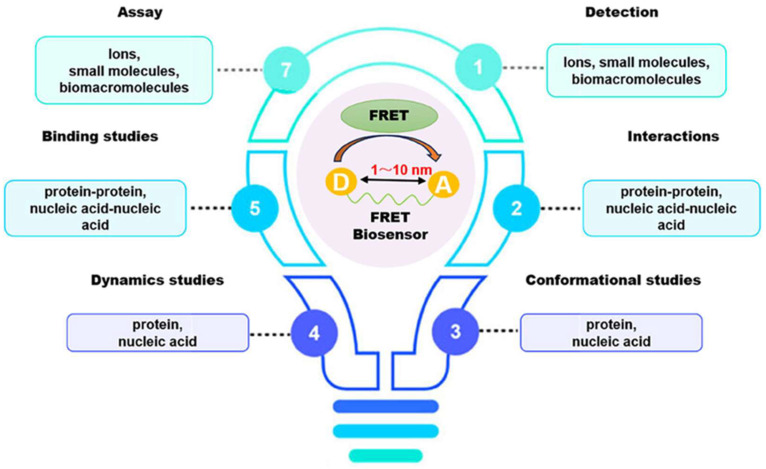
Application of FRET-based assays and biosensors in different analytical studies. Adopted with permission from ref. [12].

In recent decades, FRET has become essential for accurate nanoscale measurements in biomedical and clinical research [7,8]. FRET assays utilizing organic dyes (Figure 2) are advantageous owing to their ease of preparation, cost-effectiveness, and wide variety of applications. However, these dyes have limitations, such as weak signals, low resistance to photobleaching, short fluorescence lifetimes, poor chemical stability, and toxicity, which make them unsuitable for use inside cells [9]. FRET assays using fluorescent proteins allow for the investigation of complex and dynamic intracellular molecular interactions. However, spectral interference may limit their applications because of their broad excitation/emission wavelengths and large size [10]. Therefore, the development of novel biosensor probes is essential to overcome these challenges. Progress in nanoscience has led to the creation of engineered nanoparticles with unique optical characteristics, facilitating the use of FRET in medical and biological applications. Nanoparticles have optical and electronic properties distinct from those of larger materials, often exhibiting quantum size effects that allow tunable optoelectronic features through size and shape manipulation [11]. Fluorescent nanoparticles, such as semiconductor-based quantum dots, graphene quantum dots, and upconversion nanoparticles, have attracted attention as photostable fluorescent probes suitable for use as FRET donors. Larger nanoparticles possess electronic properties that enable superior quenching capabilities (e.g., AuNPs and GO), which have been proven to be effective fluorescence quenchers [11]. The high surface-to-volume ratio of most nanoparticles allows for the simultaneous attachment of various biomolecules, facilitating what are called “single-to-multiple” FRET donor–acceptor models [12]. Replacing organic fluorescent dyes with nanoparticles in typical FRET systems can improve energy transfer efficiency, extend working distance, and adjust spectra, thereby reducing donor–acceptor crosstalk. Consequently, FRET systems based on nanoparticles have significant potential for understanding various biological phenomena, including immunological analysis, cancer cell detection, nucleic acid analysis, and targeted drug delivery, as shown in Figure 3 [12].

In FRET-based biosensing, molecular spacers ensure optimal donor and acceptor positions for efficient energy transfer [13]. Investigating synthetic polymer spacers in FRET has revealed key factors: (i) polymer spacers control fluorophore separation, keeping them within the FRET range. FRET is ineffective if the fluorophores are too far apart, whereas very close positioning can cause quenching or signal interference. (ii) Polymers can be designed with flexible or rigid backbones, depending on the biosensor requirements. Flexible polymers allow dynamic conformational changes to detect molecular interactions, whereas rigid spacers maintain a consistent distance. (iii) Polymer spacers were selected to avoid disrupting the biological system, with common choices being polyethylene glycol (PEG) and poly (lysine). (iv) Biosensors can be tailored for high specificity by adjusting the length, flexibility, and chemical properties of the polymer spacer. In protein–protein interaction studies, spacers optimize the fluorophore distance based on the anticipated protein conformational changes [13,14,15]. (v) The appropriate spacer length and composition can enhance biosensor precision and selectivity.

This article explores the latest advancements and future potential of various FRET probes that utilize polymer-stabilized metal nanoparticles, along with the role of synthetic polymers as spacer molecules in nanoparticle stabilization [13,14,15]. This underscores the broad range of applications of FRET technology in elucidating various nanotechnology-based biosensing processes. Our analysis of the diverse properties and potential applications of polymer-stabilized AuNPs highlights their crucial role in nanoscience, life sciences, and nanotechnology. This review provides insights for future research by examining the unique features of FRET sensing using polymer-stabilized AuNPs. Additionally, it aims to provide readers with a thorough understanding of stabilization methods, thereby encouraging further research.

## 2. Molecular Spacers in Fluorescence Resonance Energy Transfer

In FRET experiments, molecular spacers are often employed to control the distance between donor and acceptor molecules [16]. These structural components are vital for positioning the donor and acceptor at the optimal distance and orientation required for FRET. Polypeptides, polymers, and other organic molecules maintain a specific gap between fluorophores, allowing precise control over the energy transfer efficiency. The FRET (energy transfer efficiency) refers to the fraction of energy transferred from donor molecules to acceptor molecules, and serves as an important measure of the proximity and interaction of molecules within analytical systems. This energy transfer is influenced by several factors, including the distance between the molecules, the overlap of their emission and absorption spectra, and their relative orientation. By modifying the length, composition, and flexibility of spacers, researchers can fine-tune the distance and orientation of donor and acceptor molecules [17]. This section aims to elucidate the functions, diversity, and design considerations of molecular spacers used in FRET systems. Therefore, selecting and designing appropriate spacers is essential for achieving the desired FRET efficiency. Thus, based on the above considerations, spacers can be of different types, as described in the following section.

### 2.1. Type of Molecular Spacers, Mechanisms, Advantages, and Limitations

#### 2.1.1. Rigid Spacers

**Figure 4:**
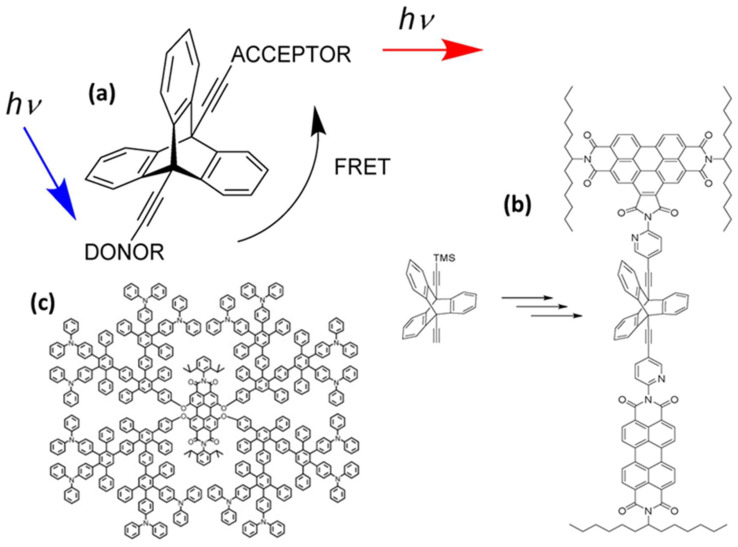
Some examples of rigid spacers. (**a**) bicyclo- [2.2.2] octane cage spacer, (**b**) triptycene as the spacer between the chromophores, and (**c**) shape-persistent polyphenylene dendrimers. Adopted from refs. [20,21].

Rigid spacers are vital components in the fields of chemistry, biochemistry, and biophysics, particularly in areas such as FRET, molecular recognition, protein folding studies, and drug development. They maintain a consistent distance between functional groups, molecules, and atoms, while restricting their relative motion [18,19]. This rigidity is essential for precise spatial control, making these spacers crucial in scenarios that require specific orientations or distances between interacting elements. Unlike flexible spacers, rigid spacers are constructed from inflexible molecular structures that preserve constant separation between the fluorophores [19]. These spacers can be designed in various forms, such as aromatic rings, cyclic structures, and other organized molecular configurations, effectively limiting movement at the molecular level. As an example, a previous study examined the impact of the geometry factor κ on FRET efficiency using benzoperylene and perylene dyads connected by a bicyclo- [2.2.2] octane cage spacer (Figure 4a) [20]. The electronic transition moments were aligned perpendicularly to theoretically inhibit energy transfer, in accordance with Förster’s theory. Surprisingly, energy transfer was not prevented, which was attributed to the molecular vibrations. Even after increasing the rigidity of the spacer with benzo groups and using highly rigid triptycene, the energy transfer persisted (Figure 4b). These findings suggest that strongly coupled molecular framework vibrations play a significant role in energy transfer, whereas molecular flexibility appears to be less important [20]. Further, intramolecular energy transfer was monitored by creating a donor–spacer–acceptor system using rigid polyphenylene dendrimers as a linker between the perylene-tetra-carboxydiimide core and peripheral triphenyl amino groups (Figure 4c) [21].

**Figure 5:**
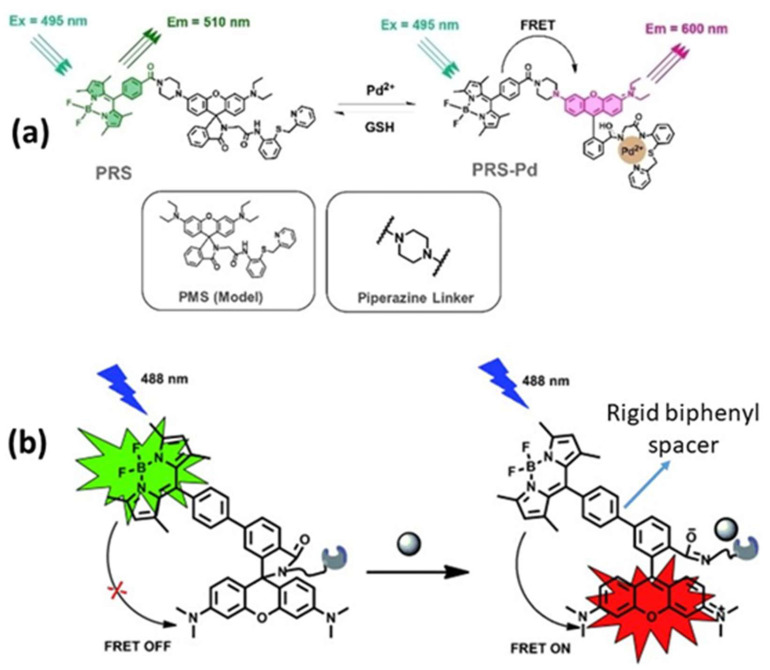
(**a**) Piperazine and (**b**) biphenyl spacers. Adopted with permission from refs. [15,25].

Rigid spacers can be organized according to their chemical structures. Commonly employed rigid spacers include aromatic rings such as biphenyl, naphthalene, and phenyl groups; these structures provide stiffness and a consistent configuration, maintaining fixed distances between fluorophores [15]. For example, a highly efficient (>99%) intramolecular FRET cassette known as the BODIPY-rhodamine platform (BRP) was developed by linking a boron dipyrromethene (BODIPY) donor to the 5′ position on the tetramethyl rhodamine (TMR) acceptor [15]. This FRET system benefited from two primary factors: the excellent degree of spectral overlap between BODIPY emission and TMR absorption, and the inclusion of a rigid and compact biphenyl spacer, which facilitated efficient through-bond energy transfer [15]. Aromatic ring spacers have been utilized in molecular recognition systems, FRET-based assays, and supramolecular structure construction. Their conjugated π-systems enable interactions such as π–π stacking, enhancing the stability of molecular assemblies [22]. Other commonly employed rigid spacers include cyclic compounds such as cyclohexane, cyclopropane, and related molecules, which establish precise geometric distances between the fluorophores [23]. These are often used in medicinal chemistry to constrain molecules into specific conformations and in the development of nanoscale molecular switches [23]. Another category of commonly employed rigid spacers includes aliphatic and saturated hydrocarbon chains. While generally flexible, certain saturated aliphatic hydrocarbons can exhibit rigidity through steric hindrance, ring closure, or other structural modifications [24]. They are used when a balance between flexibility and rigidity is required. Rigid aliphatic chains are employed when moderate flexibility is necessary. For example, adamantane, a highly rigid polycyclic hydrocarbon structure, is used in molecular recognition and drug delivery systems to ensure proper component orientation [24]. Recently, Tang et al. developed rhodamine-derived fluorescent sensors to detect Pd^2+^ ions in aqueous solutions [25]. Two sensors were created: PMS, a rhodamine-based detector, and PRS, which combines rhodamine with BODIPY to form a FRET pair. Both sensors included a piperazine linking unit and an O-N-S-N pod and ligand designed to identify and capture Pd^2+^ ions [25]. An additional category of commonly employed rigid spacers includes polycyclic aromatic hydrocarbons (PAHs), which are rigid structures composed of multiple fused benzene rings (Figure 5). These materials exhibit structural stability, rigidity, and conjugated electronic properties. PAHs are used in organic semiconductors, molecular electronics, and systems that require rigid structural scaffolding, such as FRET-based sensors and materials. Anthracene is a three-ring PAH that is often used in electronic devices and molecular sensors, where rigidity and electronic properties are critical parameters. The role of spacers with aromatic rings in regulating FRET dynamics has been well documented. These findings provide information to facilitate the development of innovative sensors and molecular structures with precisely controlled energy transfer properties.

#### 2.1.2. Advantages of Inflexible Spacers

The inflexibility of these spacers is essential for maintaining a uniform gap between the donor fluorophore and acceptor fluorophore or functional groups, which is critical for successful energy transfer and supports numerous applications. These rigid spacers provide structural stability to the molecular systems, preventing unwanted conformational changes that can impair their function. By ensuring the correct spacing of the functional groups, rigid spacers help avoid steric hindrance that might obstruct molecular interactions. The unchanging nature of these components ensures consistent experimental outcomes, as the relative positions of the elements remain constant. Furthermore, by offering a stable framework, these rigid spacers enhance molecular recognition by ensuring that the ligands or binding partners are ideally positioned for high-affinity interactions.

#### 2.1.3. Drawbacks of Rigid Spacers

When conformational flexibility is essential for functions such as protein folding or molecular signaling, rigid spacers can pose challenges. In biological settings, these inflexible spacers may obstruct the natural conformational shifts that are needed for protein–protein interaction or enzymatic reactions, reducing biological activity or adaptability. Rigid spacers often occupy more space than flexible spacers, limiting their use when smaller molecular sizes are needed or when spacers might disrupt normal operations. The production of rigid spacers, particularly those with cyclic structures or polycyclic aromatic hydrocarbons, is more complex than that of the flexible spacers. Furthermore, rigidity can enforce shapes that hinder the optimal binding of ligands or molecular partners, particularly where flexibility is advantageous.

### 2.2. Flexible/Polymeric Spacers

**Figure 6:**
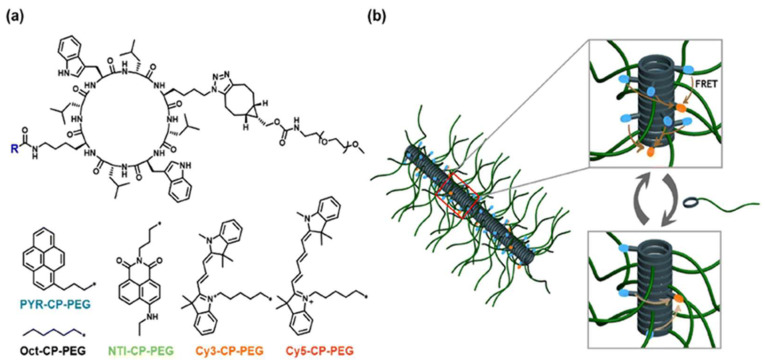
Supramolecular polymeric nanotubes with adjustable luminescence are used to create FRET systems: (**a**) molecular structures of fluorophore-cyclic peptide–polymer conjugates and spacer components; (**b**) schematic representation of modifying the luminescent properties of FRET systems using a supramolecular spacer. Adopted with permission from ref. [29].

Flexible spacers, including PEG, polyproline, and polypeptides, are integral to the development of FRET-based assays as they ensure the appropriate spacing and flexibility between the donor fluorophore and the acceptor fluorophore, which is crucial for the detection of biological processes. PEG spacers are favored in these biosensors because of their flexibility, biocompatibility, and hydrophilic nature, which enhances biosensor solubility and stability, reduces non-specific interactions, as well as improves performance. The application of PEG spacers in a field-effect transistor (FET)-based biosensor to examine lipid membrane interactions with nanoparticles, resulting in improved sensitivity and reliability, has been previously demonstrated [26]. Polyproline spacers, with their semi-rigid helical structures, influence the spatial arrangement of fluorophores in FRET systems. This rigidity is beneficial for maintaining specific distances and orientations between the donor molecules and acceptor molecules, thereby affecting the FRET efficiency [27]. Research has shown that the length and flexibility of spacers, including polyproline, significantly affect the dynamics of DNA crossover structures, as indicated by single-molecule FRET experiments (Figure 6) [27,28]. Polypeptide spacers are adaptable through the incorporation of customizable amino acid sequences for various applications [28]. The selection of amino acids and spacer length can be optimized to obtain the desired flexibility and functionality in FRET experiments. For example, cyclic peptide–polymer conjugates that self-assemble to form supramolecular polymeric nanotubes offer versatile frameworks for constructing supramolecular FRET systems [29]. The emission ratio between the monomers and excimers of PYR-CP-PEG, the FRET donor, can be modulated by incorporating a spacer into the supramolecular structure, imparting customizable and responsive characteristics to luminescent FRET systems [29]. Selecting appropriate flexible spacers is vital for designing effective FRET-based biosensors. These spacers provide the necessary flexibility between fluorophores, thereby enhancing their ability to monitor dynamic processes and conformational changes in biological systems [30]. Flexible linkers in single-molecule FRET offer insights into biomolecular conformational dynamics and functional mechanisms.

## 3. Surface Functionalization Strategies for Gold Nanoparticles

**Figure 7:**
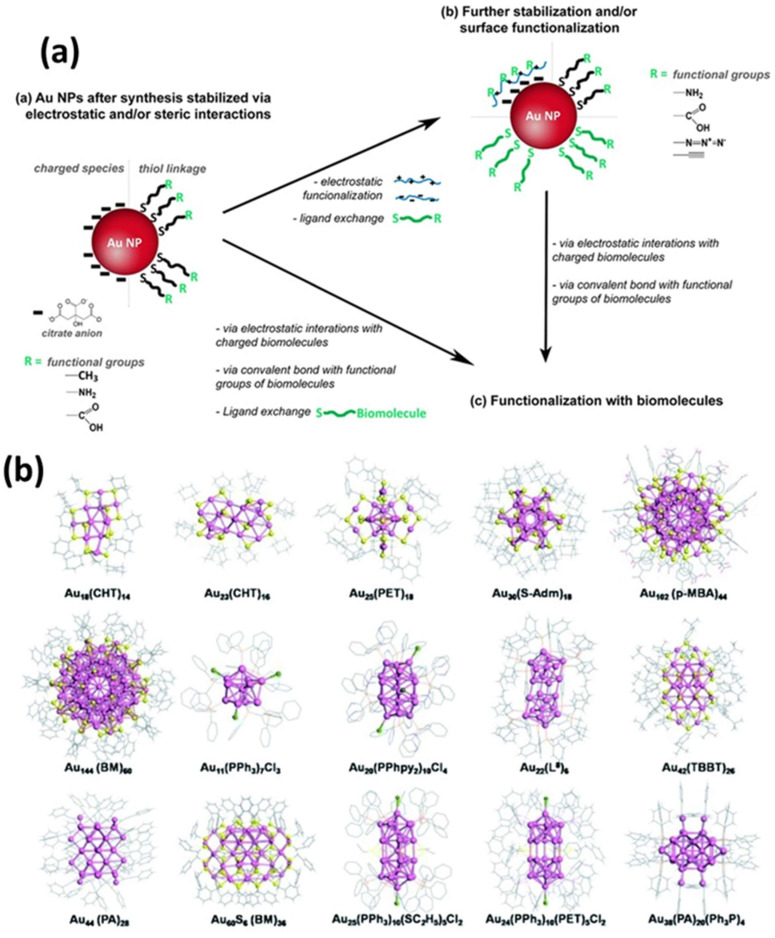
(**a**) Schematic illustration of the processes for stabilizing and/or modifying the surface of AuNPs, followed by surface modification. (**b**) Illustrative crystal structures of AuNCs encapsulated by various ligands synthesized through the reduction in the gold salt (complex). The ligands include: cyclohexanethiol (CHT-H), phenylethanethiol (PET-H), adamantanethiol (H-S-Adm), p-mercaptobenzoic acid (p-MBA-H), benzyl mercaptan (BM-H), triphenylphosphine (PPh3), bis (2-pyridyl)-phenylphosphine (PPhpy2), 1,8-bis(diphenylphosphino) octane (L8), 4-tert-butylbenzenelthiol (TBBT-H), phenylacetylene (PA), and ethanethiol (H-SC_2_H_5_). Adopted with permission from refs. [52,53].

**Figure 8:**
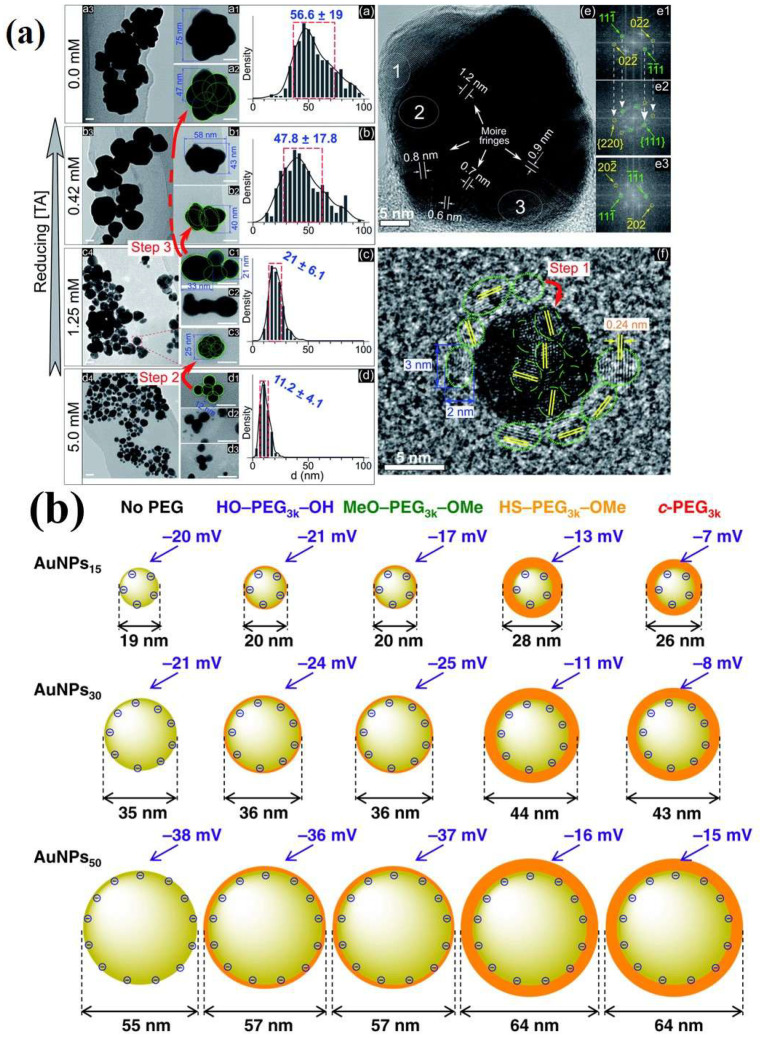
(**a**) (**a**–**d**) TEM images at varying magnifications (all scale bars—20 nm) and size distributions of GNPs in solutions with concentrations of 0.0, 0.42, 1.25, and 5 mM terephthalic acid (TA). Structures in which larger particles emerge from the aggregation of smaller particles are marked by green circles and numbers. The red arrows indicate the progression of aggregation from smaller to larger particles. The histograms display blue numbers indicating the mean ± standard deviation of the size distribution; (**e**) HRTEM of a superimposed GNP (45 nm) in a solution devoid of TA; (**e1**–**e3**) electron diffraction patterns derived from the Fast Fourier Transform (FFT) obtained from selected areas (1, 2, and 3 in (**e**)) illustrating single crystals (**e1**,**e3**) and a superimposed crystal (**e2**). The white arrows in (**e1**,**e2**) highlight the repetition of the Miller indices from (**e1**) in (**e2**). (**f**) HRTEM image of an isolated GNP (10 nm) in a solution without TA, encircled by smaller GNPs measuring 2–3 nm in diameter (green dotted circles). Adopted with permission from ref. [56]. (**b**) effect of PEG on DLS size and ζ-potential of gold nanoparticles stabilized with various chemically modified PEG molecules (AuNPs/No PEG, AuNPs/HO–PEG_3k_–OH, AuNPs/MeO–PEG_3k_–OMe, AuNPs/HS–PEG_3k_–OMe, and AuNPs/*c*-PEG_3k_) and different molecular weights of PEG. Adopted with permission from ref. [68].

To achieve greater efficiency and sensitivity, researchers are exploring alternative donor and acceptor materials to serve as replacements for conventional organic dyes. Various nanoparticles (e.g., graphene quantum dots (GQDs), quantum dots (QDs), and upconversion nanoparticles (UCNPs) have demonstrated potential as FRET donors [31,32,33]. These materials are appealing because of their attributes, including enhanced efficiency, increased stability, and improved performance in biosensing applications. Among the available nanoparticles, AuNPs are notable for their unique characteristics, such as surface plasmon resonance (SPR) effects, high conductivity, ease of surface functionalization, and exceptional fluorescence-quenching capabilities [34,35]. Gold nanoclusters (AuNCs) have been synthesized with chemical methods that have been traditionally used for AuNPs (≥2 nm) by employing Au precursors with stabilizing agents. By controlling the reaction kinetics, researchers can create smaller AuNPs that are approximately 2 nm in size, which are now classified as AuNCs [36]. These methods can produce AuNCs with narrow size distributions; however, achieving atomic-level monodispersity without further purification remains difficult [37]. AuNCs are distinct entities, with stability determined by specific molecular configurations based on “magic numbers” of Au atoms and selected surface ligands [38]. Recent studies have identified phosphines, thiols, and amines as the most common functional groups used for ligand nucleation and stabilization during synthesis. These groups interact strongly with gold atoms in addition to maintaining stability and solubility in a range of aqueous and non-aqueous solvents. The thiol- and thiol-derived ligands are particularly effective stabilizers for AuNCs, which range from simple molecules (such as alkane-thiols and glutathione) to more complex structures (such as bovine serum albumin and thiolated polyethylene glycol) [39,40,41,42,43,44,45,46,47,48,49,50,51]. Utilizing biological molecules, especially short peptides and proteins, as templates for AuNCs stabilization is a highly effective strategy for imparting functionality and addressability during synthesis. Another approach involves the kinetic assembly of metal ultra-clusters to engineer a high metal content by manipulating electrostatic interactions, van der Waals interactions, and steric interactions (Figure 7) [52,53]. For instance, modifying the pH value or ionic strength of dilute solutions containing citrate-capped AuNPs (2–100 nm in size) decreases electrostatic repulsion, resulting in agglomeration [54,55]. AuNPs with lysine or cysteine caps also form reversible aggregates at high concentrations when charge changes occur owing to pH shifts [56]. Interestingly, in a straightforward method for controlling the size of AuNPs synthesized via plasma–liquid interfaces, the addition of ligands to the precursor solution interrupts the rapid growth of AuNPs in distinct phases [56]. By adjusting the ligand concentration, the size of the AuNPs can be regulated, corresponding to the reciprocal functions of the concentration. Surface analysis revealed that ligand adsorption on the AuNPs prevented them from merging into larger particles. The effectiveness of ligands in size regulation is determined by their affinity for the AuNP surface, which is ranked as thiol > amine > carboxylate (Figure 8a) [56].

Nonetheless, these techniques frequently lead to uncontrolled expansion, resulting in uneven aggregates that may exceed more than a hundred nanometers in size [57,58,59,60,61,62,63,64]. This phenomenon poses a challenge in the assembly process; the processing goal involves finding the right balance of stabilizers to stop growth, while forming small clusters under 100 nm with high metal content for improved functionality. These ligands can be linked to nanoparticles through surface functional groups, imparting a charged exterior to the nanoparticle, which induces coulombic repulsion and enhances nanoparticle dispersion. However, when the charge screening becomes significant, salts can reduce this repulsion, causing NPs precipitation or “salting-out” [65]. The stability of nanoparticles coated with citrate [66], orthophosphoric acid [67], and other proton-exchanging compounds depends on the pH value of the medium, which directly affects the ζ potential. Nanoparticle aggregation occurs when the magnitude of ζ-potential decreases below pH values near the pKa associated with the surface functional groups (the level of effective coulombic repulsion) as demonstrated in Figure 8b [68]. Anionic ligands, including citrate and phosphate, are effective in stabilizing nanoparticles in basic environments or mildly acidic environments [69]. In contrast, cationic ligands such as alkyl ammonium are more suitable for stabilization in environments ranging from acidic to slightly alkaline. Despite the presence of stabilizers on the surfaces of colloidal AuNPs, further surface modifications are required for various applications [67]. Surface functionalization is essential because it facilitates interactions between AuNPs and their surrounding environment, mainly through functional groups such as carboxyl or amine moieties [67]. Another approach to ionic stabilization involves the formation of a steric barrier in order to prevent aggregation. This steric stabilization can be accomplished by either surrounding the NPs with a ligand shell or embedding the NPs within an inorganic matrix or a polymeric matrix. Polymers function as effective stabilizing agents by expanding the hydrodynamic radius of nanoparticles, thereby preventing the metal cores from coming into direct contact with the medium [70]. While advantageous for in vivo applications that require prolonged circulation, this approach may impede swift diffusion into extravascular spaces. Therefore, the size of NPs is an important parameter for biodistribution applications [71]. Various water-soluble polymeric ligands are frequently derived from PEG and carbohydrates, including starch [72], dextran [73], and chitosan [74]; some commonly used polymers for gold nanoparticle stabilizations are provided in Table 1 [75,76,77,78,79,80,81,82,83,84,85,86,87,88,89,90,91,92,93,94,95,96,97,98,99,100,101,102,103,104,105,106,107,108,109,110,111,112,113,114,115,116,117,118,119,120,121,122,123].

**Table 1:** Commonly used polymeric stabilizers for AuNPs/NCs. (* in figures representing successive monomer units).

Polymer	Function	Chemical Structure of Polymer	References
Poly-amidoamine (PAMAM)	Amine groups and amide groups. Dendrimer imparts a steric framework.	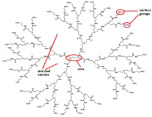	[75,76,77,78,79,80]
Poly(3,4-ethylene dioxythiophene)	Reducing and immobilizing agent	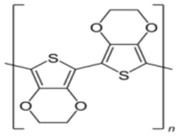	[81,82,83]
Trioctylamine	Stabilizers	** 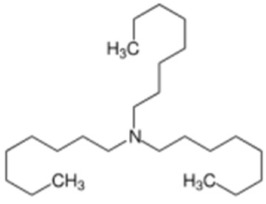 **	[83]
Poly(N-vinylpyrrolidone) (PVP)	Steric stabilization	** 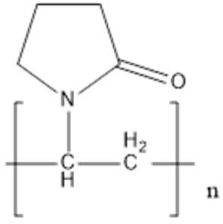 **	[84,85,86,87,88,89,90]
Poly(ethylene) glycol (PEG)	Stabilizer	** 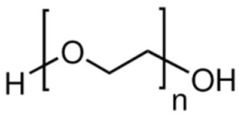 **	[91,92,93,94]
Poly(vinylcaprolactame) (PVCL)	stabilizer	** 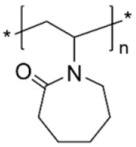 **	[85]
Poly(diallyldimethylammonium chloride) (PDADMAC)	Polycationic stabilizer	** 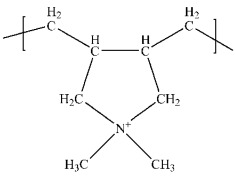 **	[95]
Polyethyleneimine (PEI)	Cationic stabilizer	** 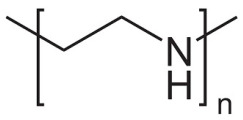 **	[96,97,98,99,100,101,102]
Polyvinyl alcohol	Reducing and stabilizing agent	** 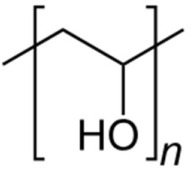 **	[103,104,105,106,107]
Poly[2-hydroxy-3-(naphthalen-1-ylamino)propyl methacrylate] (PHNA)	Stabilizing agent	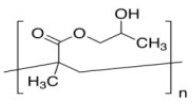	[108]
Chitosan	Stabilizing agent	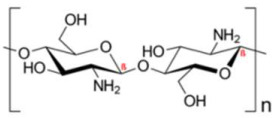	[109,110,111,112,113,114,115,116,117]
3-Aminopropyltrimethoxy silane (3 APTMS)	Stabilizing agent	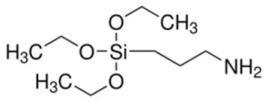	[118,119]
Pullulan	Stabilizing agent	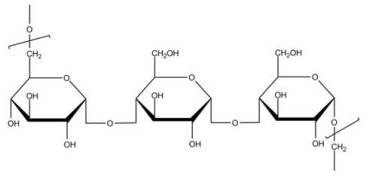	[120]
Pectin	Stabilizing agent	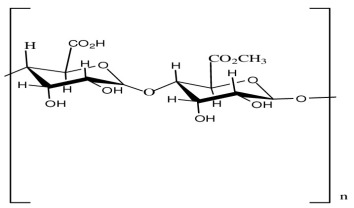	[121]
Polyaniline	Stabilizing agent (Conducting polymer)	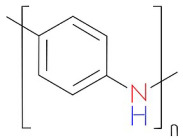	[122]
Poly-Indole	Stabilizing agent (Conducting polymer)	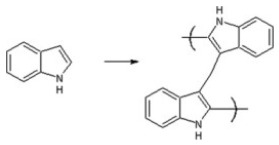	[123]

Figure 7 illustrates three distinct strategies for synthesizing polymer/Au nanocomposites, each with its own benefits and limitations. The grafting process, also termed post-modification, is the most commonly utilized method because it involves the separate synthesis of polymer and inorganic components. This approach enables precise regulation of the physical attributes of the nanoparticles (e.g., size and shape) as well as the chemical properties of the polymer (e.g., structure, molecular weight, and chemical composition) before they are integrated into the nanocomposite [124]. However, a potential drawback of this technique is its limited grafting density, particularly when dealing with polymers of high molecular weight [125]. As previously discussed, chemisorption of pre-synthesized polymers onto AuNPs can be facilitated through the thiol moiety by either removing the reversible addition–fragmentation chain-transfer (RAFT) molecule [126,127,128,129] or retaining it. RAFT functionalization of AuNPs provides a versatile strategy for creating durable hybrid nanomaterials with superior properties. This approach uses RAFT polymerization to synthesize polymer chains that anchor to the surface of AuNPs, resulting in a polymer-coated “core–shell” configuration for various applications. The functionalization process combines RAFT polymerization control with the strong affinity of gold for sulfur atoms. Most RAFT agents are thiocarbonylthio compounds containing sulfur atoms that form robust covalent bonds with the gold surface, ensuring stable polymer-nanoparticle connections [128]. For instance, hydrophilic polymers synthesized via RAFT polymerization have been used to functionalize AuNPs [128]. In 2010, Davis et al. showed that AuNPs can be stabilized using polymers that react to variations in temperature and pH levels. RAFT polymerization was employed to produce these polymers, which consisted of poly(2-aminoethylmethacrylamide), poly(N,N-dimethylaminoethyl acrylate), poly(acrylic acid), poly(oligoethylene oxide acrylate-co-diethylene oxide acrylate), poly(oligoethylene oxide) acrylate, and poly(N-isopropyl acrylamide) [130]. Similarly, Destarac et al. applied the MADIX/RAFT polymerization approach to coat AuNPs with three different polymers, each of which demonstrated distinct behavior in water: a thermoresponsive polymer (PNiPAM), a cationic polymer (poly(3-acryl-amidopropyl) trimethylammonium chloride]), and a pH-responsive polymer (PAA) [131]. Poly(N-vinyl caprolactam) was also evaluated [132]. The optical properties of these nanocomposites were subjected to variations in pH and temperature, which were contingent on the specific polymer that was utilized. In 2010, Klok et al. successfully synthesized AuNPs with a size ranging from 5 to 47 nm. These particles were enveloped in polyethylene glycol methacrylate in an aqueous medium and exhibited thermoresponsive characteristics [133]. In 2011, Klok et al. described the assembly of a series of AuNPs (12, 28, and 51 nm) with varying chain lengths by modifying poly(pentafluorophenyl methacrylate) post-polymerization, which was produced via RAFT polymerization [134]. Following post-polymerization modification, researchers prepared nanocomposites with a range of surface chemical properties, including charge and polarity, which were subsequently utilized as optical sensors for examining various types of biologically important media. In 2013, Vana et al. applied a PNiPAM coating with one or more trithiocarbonate (TTC) groups to AuNPs [135]. They described the important role of the TTC groups in ensuring attachment to the gold core, thereby enhancing the stability of the nanocomposite. It is interesting to note that polymers with several TTC groups preserved a consistent spacing among the Au cores, even if polymers that exhibited higher molecular weights were employed. Vana et al. explored a grafting approach to develop other controlled nanostructures with Au [136,137].

## 4. Polymer-Stabilized Gold Nanoparticles in Fluorometric Sensing

AuNPs are outstanding energy acceptors for use in biosensor technology because of their unique electrodynamic properties, which provide well-defined plasmon resonance. This resonance can be spectrally aligned with the emissions from various donors [138]. The use of fluorescence quenching by AuNPs has significantly expanded the range of optical probe development techniques in applications such as biology, theragnostic, and imaging [139,140,141,142,143]. A consideration of the energy transfer mechanism is crucial for the advancement of distance-dependent optical biomolecular detection methods. Theories such as FRET, nanosurface energy transfer (NSET), and Inner filter effect (IFE) are instrumental for understanding the quenching mechanism of AuNPs [144,145,146,147,148,149,150]. However, in this article, we are focusing on the suitability of polymer spacers in FRET-based sensing systems, although some of the recent advancements in the field are provided in Table 2.

**Figure 9:**
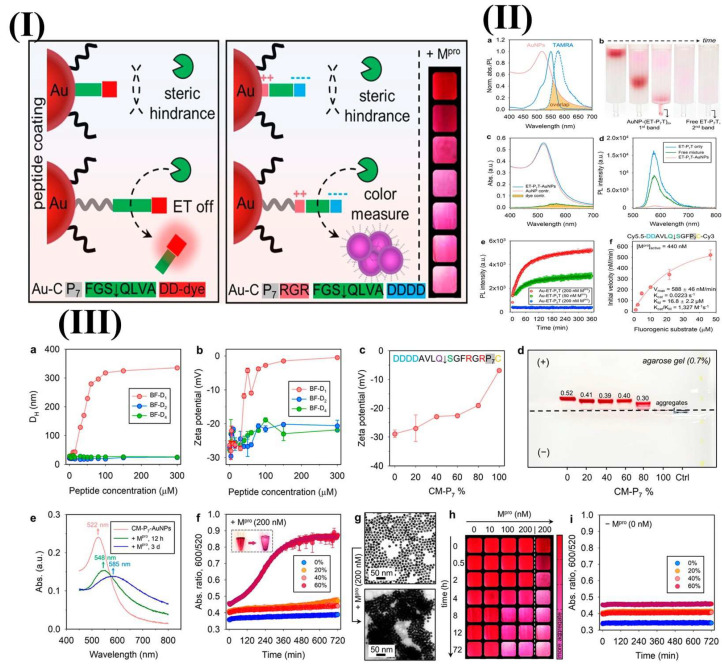
(**I**) The creation of modular peptides for the energy transfer and colorimetric detection of SARS-CoV-2 M^pro^ necessitates a spacer domain to decrease steric hindrance, thereby facilitating the proteolytic activation of the optical signals. This is pertinent to nanosurface energy-transfer sensors that employ AuNP–peptide–dye configurations and colorimetric sensors with zwitterion peptide-coated AuNPs. The integration of polyproline improved the activation of NSET sensors by M^pro^. (**II**) (**a**) The normalized optical spectra of AuNPs and TAMRA dye demonstrate spectral overlap, with solid lines indicating absorption and dashed lines representing photoluminescence (PL). (**b**) Time-lapse images from size-exclusion chromatography illustrate the purification process of AuNP conjugates from the free peptide–dye. (**c**) The absorption spectral deconvolution of AuNP–(ET-P_7_T) n conjugates revealed contributions from both AuNPs and dyes. (**d**) PL spectra are provided for free ET-P_7_T peptide, AuNPs combined with ET-P_7_T peptide, and AuNP–ET–P_7_T conjugates. (**e**) Time-dependent PL variations for AuNP–(ET-P_7_T) 257 and AuNP–(ET-P_0_T) 241 conjugated with M^pro^. (**f**) kcat/Km for ET-P_7_ hydrolysis by M^pro^. (**III**) Activation of colorimetric sensors by Mpro. The hydrodynamic radii (**a**) and zeta potential value (**b**) of citrate–AuNPs that are mixed with back filler peptides at different concentrations. Zeta potential (**c**) and agarose gel (**d**) AuNPs with 0–100% CM-P_7_. (**e**) The absorption of 60% CM–P_7_–AuNPs with M^pro^ showed red shifts in the SPR band. (**f**) Demonstrating time progression of the ratio metric absorbance of CM-P_7_–AuNPs with M^pro^. (**g**) TEM images depict monodispersed CM–P_7_–AuNPs and gold aggregates after proteolysis. (**h**) The concentration of M^pro^ and the time-dependent color evolution of CM–P_7_–AuNPs are depicted. (**i**) The control monitored the time-dependent ratio metric absorbance values of CM-P_7_–AuNPs without M^pro^. Adopted with permission from ref. [153].

An innovative sandwich FRET biosensor was developed to detect lysozyme using peptide-functionalized AuNPs (pAuNPs) and a FAM-labeled aptamer (Apt-FAM) [151]. When Apt-FAM and pAuNPs were combined, they exhibited partial fluorescence quenching via an inner filter effect, with minimal energy transfer due to electrostatic repulsion. The presence of lysozyme induced the binding of Apt-FAM and pAuNPs, creating a pAuNPs-LYZ-aptamer sandwich structure [151]. This interaction brings the pAuNPs and Apt-FAM into close proximity, enabling FRET and leading to the quenching of FAM fluorescence [151]. In another study, a fluorescence spectrometric sensor detected Hg^2+^ in water using AuNPs functionalized with thiolated DNA. This sensor leveraged the conformational change in ssDNA triggered by Hg^2+^, which enhanced FRET between fluorescein and AuNPs. Upon introduction of Hg^2+^, the ssDNA formed a hairpin structure through thymine-Hg^2+^-thymine (T–Hg^2+^–T) coordination, leading to reduced fluorescence compared to its random coil form [152]. The assembly of nanoparticles for plasmonic coupling is a common technique that is used in bioanalytical sensors. Jin et al. designed a ligand entirely composed of peptides, which included a polyproline spacer, an anchoring group, biomolecular recognition elements, and zwitterionic domains, to functionalize AuNPs as a colorimetric enzyme sensor [153]. This study indicates the role of the polyproline component, which facilitates the recognition of peptide ligands on nanoscale surfaces by the SARS-CoV-2 main protease (M^pro^), leading to plasmonic coupling through coulombic interactions [153]. The aggregation of AuNPs is driven by a reduction in the surface potential because of the enzymatic exposure of the zwitterionic module. Consequently, this system facilitates visual detection of Mpro. In contrast, no proteolysis was observed on AuNPs that were modified using a control ligand that lacks a spacer domain [153]. The NP–peptide construct was designed to detect SARS-CoV-2 Mpro using two strategies: an energy transfer (ET) AuNP–peptide–dye model and a color-based AuNP–zwitterion–peptide conjugate (Figure 9) [153]. The peptide consisted of a neutral spacer, surface anchoring group, recognition element, and amphiphilic domain [153]. A fluorescence sensor stabilized by protamine (AgNCs) was engineered to identify trinitrotoluene (TNT); this approach utilized the concept of aggregation-induced emission enhancement [154]. The interactions that involve the non-fluorescent Meisenheimer anion associated with TNT and the amino groups of the weakly fluorescent protamine result in the aggregation of PRT-AuNCs, which in turn enhances the fluorescence intensity, which is marked by a Stokes shift (λex = 300 nm, λem = 600 nm). The fluorescence intensity showed a linear increase with the presence of TNT, reaching a detection threshold of 12.44 µg/L [154].

**Figure 10:**
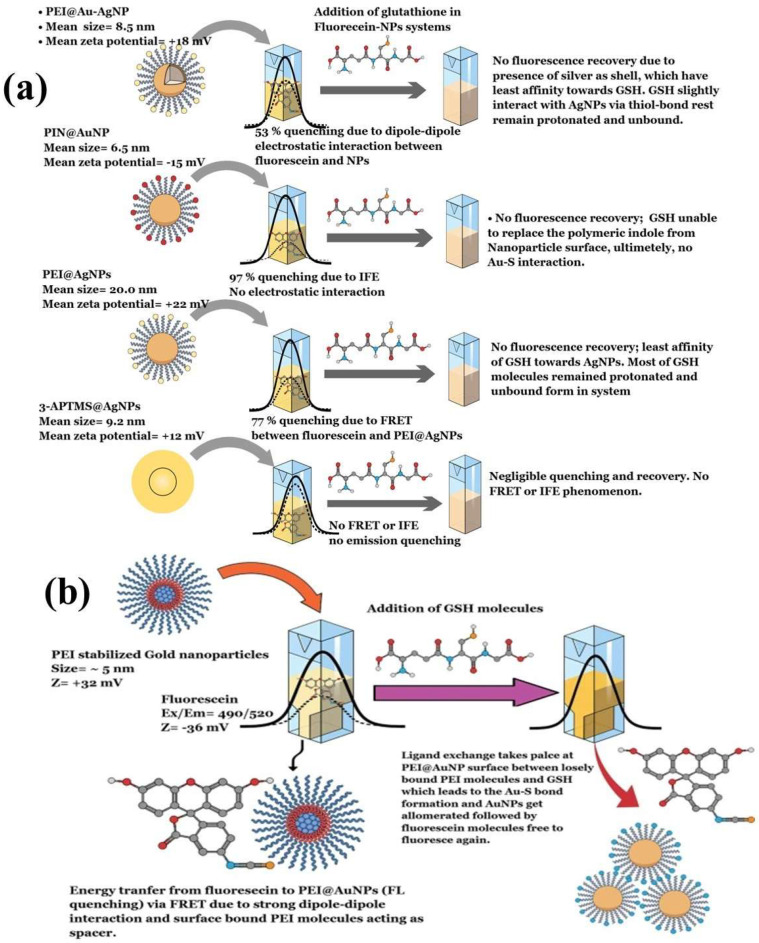
(**a**) Schematics demonstrating various AuNP–fluorescein complex formation and resulting fluorescence quenching and de-quenching mechanisms. (**b**) Fluorescein–PEI@AuNP complex, followed by associated FRET dynamics after addition of GSH. Reproduced with permission from ref. [100].

In 2012, Cepraga developed a water-soluble hybrid nano-object for fluorescence imaging by connecting two-photon chromophore-polymer conjugates to AuNPs [155]. The process involved two main steps: first, creating water-soluble chromophore-polymer conjugates, and second, attaching these conjugates to 20 nm AuNPs. The chromophore-polymer conjugates were prepared by combining hydrophobic chromophores with linear water-soluble copolymer chains via RAFT polymerization [155]. These conjugates varied in polymer length and structure, with a controlled number of chromophores per chain (1 to 21). When grafted onto 20 nm AuNPs, the nano-objects achieved a grafting density of approximately 0.5 chains/nm^2^. The polymer chain length determined the gap between the chromophore and the AuNP surface. With an increase in the average molecular weight of the polymer, the polymer corona became thicker, which affected the fluorescence characteristics due to the altered distance [155]. Similarly, Zhang et al. (2025) synthesized gold nanoclusters stabilized with tryptophan (Try@AuNCs) via chemical reduction. These AuNCs exhibited fluorescence emission at 448 nm on excitation at 368 nm [156]. The Try@AuNCs acted as a fluorescence turn-off nanoprobe in the presence of alizarin due to the inner filter effect (IFE). This nanosensor showed high sensitivity for alizarin detection, with a limit of detection of 0.092 μM [156]. Another recent study investigated the dynamics of different polymers as stabilizing agents for AuNPs on the fluorescence quenching of fluorescein, which was followed by the FRET process. The AuNPs stabilized with polyethyleneimine exhibited strong fluorescence quenching (95%) of fluorescein, which was attributed to strong dipole–dipole interactions that enabled the FRET process between the AuNPs and fluorescein (Figure 10a) [100]. The study described the role of polymer thickness and fluorophore binding strength as crucial for efficient charge transfer through FRET and IFE. Furthermore, the use of different stabilizing agents resulted in distinct quenching mechanisms. Polyindole-stabilized AuNPs showed an IFE mechanism that involved 3-APTMS-stabilized AgNPs, which weakly quenched fluorescein emission due to weak dipole interactions. Interestingly, the introduction of glutathione to the fluorescein-AuNP system reinstated the emission exclusively in polyethyleneimine (PEI) stabilized gold nanoparticles (PEI@AuNPs), which was attributed to ligand exchange occurring between PEI and glutathione (GSH) on the nanoparticle surface [100]. GSH supplanted PEI owing to the robust Au-S bond, resulting in the formation of interparticle linker structures and enabling the assembly of AuNPs. This study described a turn-on fluorescence technique for detecting GSH, utilized fluorescein-PEI@AuNPs as a probe, and achieved a detection limit of 7.8 nM. Additionally, this approach enabled the precise manipulation of the shape and size of PEI@AuNPs and provided high sensitivity [100]. This study also examined how the concentration and molecular weight of PEI affected the spacer thickness and the FRET phenomenon between fluorescein and AuNPs, with the aim of enhancing the sensitivity of fluorometric GSH sensors that have potential use for biomedical applications. Additionally, PEI@AuNPs served as fluorescent probes for detecting polymyxin B (PMB) in aqueous solutions. The detection process was based on FRET, where PMB acted as the donor and gold nanoparticles served as acceptors [99]. Among the variants, PEI@AuNP-1 showed the highest sensitivity and selectivity for PMB, with a linear detection range spanning 1–6 μM and a detection limit of 8.5 nM. This study indicated the impact of the molecular weight (MW) of PEI on the nanoscale geometry of AuNPs and its influence on FRET-based PMB sensing. These results suggest that PEI with a higher MW value serves effectively as a spacer molecule in the FRET system, although further investigation is necessary to substantiate this [99].

**Table 2:** Some of the polymer-stabilized gold nanoparticles/nanoclusters used for FRET and IFE-based sensor probe development. (NA = Not applicable).

Polymeric Stabilizer	Sensing Mechanism	Analyte	Detection Limit	Quenching/ Enhancement	Quenching/ Enhancement Efficiency	pH/Temperature	Limitations	Ref.
Polyethyleneimine (PEI)	FRET	Glutathione	7.8 nM	Enhancement	95%	7.2/RT	Selectivity	[100]
PEI	FRET	Polymyxin B	8.5 nM	Enhancement	82%	7.2/RT	Interaction with other biological molecules, such as BSA	[99]
β-cyclodextrin	FRET/PET	Dopamine	20.0 nM	Quenching	Significant	8.0	NA	[157]
Bovine Serum Albumin	FRET	Dopamine	1.8 nM	Quenching	Significant	7.5	Interference	[158]
Peptides	FRET	Histone deacetylase and protein tyrosine phosphatase 1B	1 nM to 28 pM and 0.015 to 0.3 nM/0.8 pM	Quenching	Significant	8.0	NA	[159]
Poly(9,9-bis(4′-sulfnoatobutyl) fluorene-*co*-alt-1,4-phenylene)	FRET	Cysteine	25 nM	Quenching	Significant	6.0	NA	[160]
PEI	FRET	Doxorubicin	5 pM	Quenching	Significant	7.0	NA	[161]
Polydiallyldimethylammonium	FRET	Ascorbic acid	50 nM	Enhancement	Significant	7.0	NA	[161]
mCherryProtein	FRET	Melamine	28 µM	Quenching	95–97%	8.0/RT	Explored only for bio-thiols	[162]
Poly N, N-dimethylacrylamide	FRET	L-cysteine	20.0–80.0 µM	Quenching	85%	7.5/RT	In vitro application	[163]
poly(N-isopropylacrylamide-co-2-(dimethylamino)ethylmethacrylate) (P(NIPAM-co-DMA))	FRET	Hg^2+^	31 nM	Quenching	92%	6.0	NA	[164]
Polyethylene glycol (PEG)	FRET	Hg^2+^	2.24 nM	Enhancement	High	5.65/RT	NA	[165]
Poly(*N*-isopropylacrylamide)	Photoluminescence quenching	Hg^2+^	1.9 and 1.7 nM	Quenching	Moderate	7.0/RT	Not explored in biological condition	[166]
BSA	IFE	Uric acid	0.39 μM	Quenching	High	NA	NA	[167]
Antibody-tagged bovine serum albumin	IFE	Troponin I	0.51 ng/mL	Quenching	90%	7.2/RT	NA	[168]
6-Deoxy-6-mercapto-β-cyclodextrin	IFE	Chloretetracyclin	2.7 nM	Quenching	High	8.0/RT	NA	[169]
PEI-OVA-AuNCs	IFE	tetracycline	0.563 μM	Quenching	High	RT	Binding of phenolic hydroxyl and ketone carbonyl groups	[170]

## 5. Challenges That Need to Be Addressed While Choosing a Suitable Polymeric Spacer for Efficient FRET, IFE, and NSET-Based Sensors

In general, polymeric spacers associated with gold nanoparticles pose several challenges for the development of fluorescent probes; these issues include the following:

(1) Quenching effects: For example, AuNPs can diminish fluorescence, affecting probe efficiency. Balancing the distance between the fluorophore and the AuNPs is important for mitigating quenching while enhancing the signal.
(2) Stability: Establishing a stable bond between AuNPs and the polymeric spacer is difficult, necessitating strong conjugation techniques.
(3) Uniformity: Achieving monodispersity and preparing a polymer coating are challenging, which impacts probe performance.
(4) Biocompatibility: Materials must be nontoxic and biocompatible, particularly for in vivo applications, which limit polymer options.
(5) Spacer length control: Precisely designing the length of the polymeric spacers is essential for optimizing distance-dependent interactions.
(6) Surface functionalization**:** Attaching recognition elements while maintaining optical properties and stability is a complex task.
(7) Characterization: Accurately analyzing the structure and properties of the conjugate is challenging because of system complexity.
(8) Scalability: Developing cost-effective production processes is difficult, given the need for high-quality materials.
(9) Environmental sensitivity: Probe performance may be influenced by changes in pH, temperature, or ionic strength.
(10) Photo stability: Maintaining long-term stability under continuous illumination is challenging because the components may undergo photo-induced changes.
(11) Multiplexing: Creating probes that can detect multiple targets while retaining their functionality is a complex process.

Addressing these challenges is important to facilitate the development of more effective AuNP-linked polymeric spacers for applications in biosensing, imaging, and diagnostics.

## 6. Concluding Remarks

Polymer-stabilizing agents are essential for enhancing the effectiveness and dependability of AuNP-based FRET biosensors. These agents sometimes function as molecular spacers, maintaining an ideal separation distance between the AuNPs and fluorophores to ensure efficient energy transfer and enhanced sensitivity. By carefully choosing and optimizing polymer-stabilizing agents, numerous benefits can be achieved in biosensing applications, including enhanced colloidal stability of AuNPs, prevention of non-specific interactions, and precise adjustment of FRET efficiency. Moreover, polymer spacers offer biocompatible surfaces for attaching biomolecules, thereby broadening the spectrum of detectable analytes. The adaptability of polymer-stabilizing agents facilitates the development of customizable biosensing platforms with enhanced sensitivity, selectivity, and reproducibility. By modifying the polymer chain length and composition, researchers may be able to optimize the distance-dependent FRET process and reduce background noise, resulting in more precise and reliable biosensing outcomes. Additionally, employing polymer-stabilizing agents as molecular spacers opens new opportunities for multiplexed sensing and the development of more intricate nanostructures. This approach can lead to the development of advanced biosensors that are capable of simultaneously detecting multiple analytes or executing cascade-sensing reactions. In summary, incorporating polymer-stabilizing agents as molecular spacers in gold nanoparticle-mediated FRET biosensing represents a significant advancement in this domain. This approach not only improves the performance of current biosensors but also lays the groundwork for next-generation sensing platforms with enhanced sensitivity, specificity, and versatility in various biomedical and environmental applications.

## Data Availability

No new data were created in this study.
